# Getting causal considerations back on the right track

**DOI:** 10.1186/1742-7622-3-8

**Published:** 2006-07-19

**Authors:** Michael Höfler

**Affiliations:** 1 Institute of Clinical Psychology and Psychotherapy, Dresden Technical University, Dresden, Germany

## Abstract

In their commentary on my paper Phillips and Goodman suggested that counterfactual causality and considerations on causality like those by Bradford Hill are only "guideposts on the road to common sense". I argue that if common sense is understood to mean views that the vast majority of researchers share, Hill's considerations did not lead to common sense in the past – precisely because they are so controversial. If common sense is taken to mean beliefs that are true, then Hill's considerations can only lead to common sense in the simple and well-understood causal systems they apply to. Counterfactuals, however, are largely common sense in the latter meaning.

I suggest that the road of scientific endeavour should lead epidemiologic research toward sound strategies that equip researchers with skills to separate causal from non-causal associations with minimal error probabilities. This is undeniably the right direction and the one counterfactual causality leads to. Hill's considerations are merely heuristics with which epidemiologists may or may not find this direction, and they are likely to fail in complex landscapes (causal systems). In such environments, one might easily lose orientation without further aids (e.g., defendable assumptions on biases). Counterfactual causality tells us when and how to apply these heuristics.

## Background

Scientific endeavour is stimulated by researchers who not only communicate their ideas but also reflect upon them in light of the arguments of others. Different scientists have varying perspectives because of, for example, their differing areas of expertise and biographies. Varying points of view may add arguments to the debate that the originator of an idea had not initially been aware of. In this regard, I highly appreciate the thoughtful comments of Phillips and Goodman [1] on my paper [2] on Hill's [3] considerations on causality, and I am grateful for the opportunity to respond to them. Now, let us see how my ideas persist when pondered alongside Phillips and Goodman's arguments.

## Analysis

### Coming to terms with the terms

It seems that Phillips and Goodman largely agree with me that there are no such things as "causal criteria" (temporal precedence of the factor to the outcome perhaps being the exception). They would, however, be better off not to use that term except to indicate how misleading it is. Phillips and Goodman also agree with me about the usefulness of counterfactuals although they are not fully consistent in their argumentation (on page 4f. they write that "...most everyone who uses causal language is implicitly invoking the counterfactual definition...We cannot think of any use of the word 'cause' in epidemiology ... where the author seemed to have something else in mind.". On page 3, however, they assert: "...counterfactuals are, more than anything else, guideposts on the road to common sense"). We seem, therefore, to disagree in our views on the relation between "causal criteria" and counterfactuals, and I shall explain how our views differ using the same "road" metaphor that Phillips and Goodman invoke.

Causal definitions, conceptions or models have many roots, for instance, in different scientific disciplines. (I have no preference in using one term over another, and I do not believe that the debate on these terms leads us anywhere). Causal thinking is like a road system of scientific endeavour the intent of which should be to lead scientists from different disciplines throughout the centuries toward the same arrival point, namely, to sound strategies through which to derive the right causal answers in empirical research. Phillips and Goodman consider "common sense" to be the arrival point. This is unfortunate because "common sense" has two very different meanings, and it is unclear which meaning Phillips and Goodman use when they define common sense as "first- or second order logical inferences that scientists should intuitively grasp":

(1) In the philosophical tradition of John Locke common sense means input from different senses which has to be integrated [4] so that, for instance, the vast majority of researchers in a field share the same point of view.

(2) Thomas Reid [5] and G.E. Moore [6] introduced the view that common sense beliefs are true.

In terms of meaning (1) above Hill's considerations have not lead to common sense in the past. Just the opposite is the case, and it appears unlikely that this situation will change in the future because different researchers have different interests: Substantive researchers need to identify new relations and frivolously label them "causal" to succeed (e.g. in funding, a point mentioned by Phillips and Goodman), while methodologists need to scrutinise common malpractices (and many of them do not agree with one another as well). Moreover, common sense changes over time because knowledge changes as societies, politics and other factors change that influence common sense. Hence, common sense might be wrong at least at some time.

On the other hand, in terms of meaning (2) above Hill's considerations can lead to common sense only in the simple causal systems they apply to, as I have tried to demonstrate [2].

### Getting back on the right track

The roads on the way to sound causal decisions have many branch connections, some of which have converged and now lead in the same direction (e.g. Pearl [7] has shown that counterfactual models, causal graphs and some non-parametric structural equation models are equivalent for all practical issues), and some of which lead down blind alleys (e.g. some structural equation models that failed to recur in experiments [8]). During the last decades, however, the traffic has concentrated in the direction delineated by counterfactual causality. There are important wherefores, especially in epidemiology, that have been outlined elsewhere ([9] and references therein). Therefore, I believe that, yes, counterfactual causality should be used as the standard conception of causality. In epidemiology, causal decisions are inevitable (despite the Duhem-Quine problem mentioned by Phillips and Goodman). Consider the example that one has to choose between two available options for public health intervention. How can one decide without referring, at least implicitly, to a conception of causality? How can one decide without referring to assumptions, data, and models on how the outcome would turn out when using one option instead of the other (i.e., counterfactual causality)?

Therefore, I consider counterfactual causality to be common sense in the meaning of common sense as true beliefs, and it is the direction along which one approaches the target of optimal causal inference. As pointed out by Rothman and Greenland [10], making causal decisions is nothing more than an error-prone process (as is the case when measuring a condition, a point discussed by Phillips and Goodman). The aim is "simply" to minimize the probabilities to draw false positive and false negative conclusions on the existence of a causal effect. More precisely, this is a decision problem with the potential harm of each possible error resulting in consequences for individuals' health as well as for health costs.

Seemingly, and confirmed by Phillips and Goodman Hill was already propelling his ideas in the counterfactual direction but, unfortunately, he did not point that out unambiguously. In Höfler [2], I have tried to demonstrate that researchers applying his considerations often blindly followed his heuristics rather than take care to maintain the counterfactual direction toward which they shall point. The problem is that Hill's heuristics do not work well in every landscape, namely, a landscape representing a particular causal system. This is exactly the gap that I have attempted to fill.

For instance, Hill's consideration on strength of association can be viewed in light of the heuristic always to walk toward the steepest observable gradient when searching for the highest peak in misty mountains. The probability that this heuristic will fail rises with the number of peaks in the mountains. If you know the direction, you might lose it randomly (e.g., because your compass breaks), or systematically because you make wrong assumptions on how to maintain the right direction when walking around hurdles such as hills or lakes (i.e., biases).

Phillips and Goodman distinguish between counterfactuals on the one hand and hypotheticals and *ex ante *hypotheses on the other. This distinction appears artificial to me. Suppose one asks: "If the data were free of all biases (applying a particular bias model), how would the data be expected to change?" This constitutes a counterfactual difference between the (presumably) true condition of certain biases of certain magnitudes being present versus the counterfactual condition of no biases. Thus, the biases have caused a change in the data – as compared to the dataset that would emerge from a causal system free of biases (besides the unlikely possibility that the biases cancel out exactly). Counterfactuals are always hypothetical.

Phillips and Goodman provide several other relevant arguments that are not directly related to my paper. I share most of their viewpoints including those on common malpractices in data analysis, reporting of results and funding. Scientific success (in terms of impact factors, funding etc.) appears more likely if sharp conclusions such as "We have demonstrated a previously unknown effect..." are drawn rather than more careful ones, such as "Given the data and a defendable model for biases the probability of a causal effect of a magnitude of greater than *c *is X...". As long as this undesirable state of affairs remains the case, better practices are likely to remain in Phillips and Goodman's words an "esoteric sideline" of research.

## Conclusion

Counterfactuals define the direction in which one has to go to derive sound strategies to separate causal from non-causal associations. Hill's considerations are not common sense and do not lead us to common sense; they are merely heuristics with which epidemiological researchers may or may not find the right way to causal decisions with minimal error probabilities. These heuristics easily fail in complicated and poorly understood environments (causal systems), but counterfactual causality tells us which questions to pose when deciding whether to apply them or not.

## Competing interests

The author(s) declare that they have no competing interests.
